# Synergistic effects of salicylic acid based plant growth regulators on growth, photosynthetic performance, and antioxidant defense of *Polianthes tuberosa L.* under drought stress

**DOI:** 10.1038/s41598-026-59958-x

**Published:** 2026-06-30

**Authors:** Liaqat Ali, Aqib Nawaz Mughal, Muhammad Shahid Rizwan, Qaiser Shakeel, Sajid Masood, Muhammad Ali, Hamid Nawaz, Muhammad Farhan Saeed, Aftab Jamal, Árpád Székely, Majed Alotaibi, Nawab Ali, Mahmoud F. Seleiman

**Affiliations:** 1 Cholistan Institute of Desert Studies, The Islamia University of Bahawalpur, Bahawalpur, 63100 Pakistan; 2 Department of Horticulture, Regional Agricultural Research Institute, Bahawalpur, Bahawalpur, 63100 Pakistan; 3 Department of Soil Science, Bahauddin Zakariya University, Multan, 61000 Pakistan; 4 Institute of Agro-Industry and Environment, The Islamia University of Bahawalpur, Bahawalpur, 63100 Pakistan; 5 Department of Environmental Sciences, COMSATS University Islamabad, Vehari-campus, Vehari, 61100 Pakistan; 6 Department of Soil and Environmental Sciences, Faculty of Crop Production Sciences, The University of Agriculture, Peshawar, 25130 Pakistan; 7 Research Centre for Irrigation and Water Management, Institute of Environmental Sciences, Hungarian University of Agriculture and Life Sciences, Anna-Liget str. 35, Szarvas, 5540 Hungary; 8 Department of Plant Production, College of Food and Agriculture Sciences, King Saud University, P.O. Box 2460, Riyadh, 11451 Saudi Arabia; 9 Department of Biosystems and Agricultural Engineering (BAE), College of Agriculture and Natural Resources, Michigan State University, East Lansing, MI USA

**Keywords:** Drought stress, Foliar application, Reactive oxygen species, Chlorophyll fluorescence, Root architecture, Oxidative stress, Biostimulants, Physiology, Plant sciences

## Abstract

Drought stress is a major limitation to sustainable floriculture in arid regions, severely affecting plant growth and flower quality. To mitigate these effects, this study evaluated foliar application of salicylic acid (SA; 300 ppm) applied alone and in combination with indole-3-acetic acid (IAA; 200 ppm), ascorbic acid (AA; 300 ppm), and chitosan (CS; 300 ppm) on the growth, photosynthetic performance, and antioxidant defense of *Polianthes tuberosa L.* under graded drought conditions [90%, 70%, and 40% water-holding capacity (WHC)]. The experiment was arranged in a factorial randomized complete block design with five replications. Drought stress was imposed for 8 weeks until first floret opening, while foliar treatments were applied four times (25, 40, 55, and 70 days after planting) starting at the three-leaf stage (BBCH 13). Results showed that severe drought (40% WHC) stress significantly reduced shoot biomass by ~ 60%, root biomass by 65–70%, and root surface area by ~ 45%, compared with well-watered conditions (90% WHC), indicating strong growth inhibition. A significant drought × PGR interaction confirmed that treatment responses were stress dependent. Among treatments, SA + IAA most effectively improved growth, biomass accumulation, and reproductive traits across drought levels, whereas SA + CS improved plant water status and reduced electrolyte leakage. In contrast, SA + AA better maintained chlorophyll fluorescence parameters and photosynthetic efficiency under drought stress. These responses were supported by increased antioxidant enzyme activity (SOD, CAT, POD), higher phenolic content, and soluble proteins, contributing to improved drought tolerance. Overall, SA-based combinations improved drought tolerance in a trait-specific manner, with SA + IAA promoting growth, SA + AA supporting photosynthetic performance, and SA + CS improving water status and membrane stability under water-limited conditions. These findings suggest that SA-based growth regulator combinations may enhance drought resilience and sustain growth in *Polianthes tuberosa* under water-limited conditions.

## Introduction

Tuberose (*Polianthes tuberosa* L.) belongs to the family of Agavaceae, is a flowering, perennial, and bulbous plant^1^. Although tuberoses are primarily grown as ornamental flowers, they are commercially important due to their long stems, attractive floral appearance, and pleasing fragrance. Due to its high ornamental and commercial value, tuberose is widely cultivated for cut flower production and landscape use, where maintaining floral quality under environmental stress is essential^2^. In Pakistan, tuberose cultivation supports smallholder farmers and the ornamental-plant sector, contributing directly to rural livelihoods^3^. However, drought stress has emerged as one of the most critical environmental constraints limiting tuberose productivity. Water deficit conditions significantly reduce vegetative growth, spike length, floret number, and overall flower quality, thereby lowering its market value^4,5^.

Water deficit not only restricts plant growth and yield but also induces severe morpho-physiological and biochemical disruptions^6^. One of the earliest plant responses to drought stress is stomatal closure, which conserves water but limits CO₂ uptake and thus suppresses photosynthetic carbon assimilation^7^. Similar drought-induced physiological constraints have been documented in rose (*Rosa* spp.), gladiolus (*Gladiolus grandiflorus*), and lily (*Lilium* spp.) ^7–9^.

In addition to aboveground effects, drought stress significantly influences root system architecture, which is essential for plant adaptation to water-limited environments^10^. Root traits, such as length, surface area, volume, and branching, determine water and nutrient acquisition under moisture deficit^11^. Depending on the stress intensity and species plasticity, drought may suppress root growth or promote deeper root elongation to access moisture. In bulbous ornamental crops such as tuberose, root system development is particularly important because it directly supports water uptake, nutrient acquisition, and sustains flowering under stress conditions. However, despite its importance, tuberose-specific evidence on root architectural responses under drought stress remains limited, and the interaction of these traits with exogenous growth regulators is not well understood. Therefore, characterization of root morphological responses is essential for understanding drought-adaptive mechanisms in this species.

At the cellular level, drought accelerates the formation of reactive oxygen species (ROS) that act as both signaling molecules and agents of oxidative stress. Chloroplasts and mitochondria can be damaged by an overabundance of ROS that can affect membranes and inactivate enzymes, and inhibit photosynthesis^12^. To prevent this damage, plants utilize a complex antioxidant defense system consisting of both enzymatic components, such as superoxide dismutase (SOD), catalase (CAT), peroxidases (POD, APX, GPX), and dehydroascorbate reductase (DHAR) as well as non-enzymatic antioxidants such as ascorbic acid, glutathione, proline, and phenolics^13^.

Recently, the use of plant growth regulators (PGRs) or biostimulants continued to expand as environment-friendly products used to enhance plant productivity and stress tolerance^14^. These compounds, including salicylic acid (SA), cytokinins, jasmonic acid, ascorbic acid (AA), auxins such as indole-3-acetic acid (IAA), and chitosan (CS), have been identified for their positive effects on plant performance under drought stress, with respect to osmotic adjustment, chlorophyll retention, and redox homeostasis^15^.

Salicylic acid (SA) is one of the well-known plant growth regulators that has been shown to regulate root growth and development during drought stress. It increases root elongation, root biomass, and lateral root development when applied exogenously to plants under limited water conditions. For example, SA improved root system development and drought resilience in sesame^16^, and similar increases in root growth as well as osmotic adjustment have been found in fennel plants subjected to drought stress^17^. Overall, it appears that the relationships between salicylic acid and drought stress play an important role in determining root morphology, contributing to plant water uptake efficiencies and overall plant performance. However, its integration with photosynthetic electron transport processes and antioxidant regulation in tuberose under drought stress remains poorly understood.

Foliar application of PGRs has emerged as an effective strategy for inducing drought tolerance by regulating photosynthesis, water relations, and redox homeostasis. For instance, PGR-mediated enhancement of antioxidant defense and ROS homeostasis improved drought tolerance in *Catharanthus roseus* seedlings^18^. Similarly, combined application of gibberellic acid and humic acid significantly increased bud formation and flowering stem height in lily through enhanced net photosynthesis and reproductive efficiency^19^. Salicylic acid has been reported to regulate stomatal behavior and osmotic stress tolerance in tomato plants under drought conditions^20^, while methyl jasmonate improved water stress tolerance by enhancing leaf water potential, RuBP regeneration, and CO₂ assimilation^21^. Ascorbic acid plays a crucial role in maintaining membrane stability, stimulating cell division, and mitigating electrolyte leakage during oxidative stress^22^. Moreover, chitosan application has been shown to increase proline and glycine betaine accumulation in *Solanum melongena* under stress conditions, whereas IAA enhances nutrient uptake, chlorophyll biosynthesis, and the expression of stress-responsive genes under water deficit^23^.

Recent studies suggest that combining multiple growth-promoting agents can provide greater drought protection than individual treatments by targeting complementary physiological pathways^24,25^. For instance, the combined application of PGPR and ZnO nanoparticles improved drought tolerance and essential oil quality in grapefruit mint through coordinated physiological and biochemical regulation^26^. These findings highlight the potential of synergistic stress-mitigating strategies to enhance plant resilience more effectively than single-component applications.

Although numerous studies have demonstrated the beneficial effects of individual plant growth regulators on drought tolerance, knowledge regarding the coordinated effects of salicylic acid-based combinations on root architecture, photosynthetic electron transport, chlorophyll fluorescence characteristics, and antioxidant defense mechanisms in *P. tuberosa* remains insufficient. Despite increasing interest in salicylic acid-mediated stress mitigation strategies, studies evaluating such combinations under graded drought stress in tuberose are scarce, and the existing evidence is fragmented. Furthermore, the physiological relationships among root system adaptation, photosystem II efficiency (ΦPSII), linear electron flow (LEF), and antioxidant enzyme activity under drought stress have not been sufficiently characterized in this species, particularly under arid and semi-arid cultivation conditions.

In arid and semi-arid regions such as the Cholistan desert in Pakistan, tuberose is often grown with very limited water. That is why it is important to understand how this plant survives drought, so its production can be improved under water-scarce conditions. Therefore, in this study it was hypothesized that combined application of SA with auxin, antioxidant, or biopolymer-based PGRs would enhance drought tolerance more effectively than individual treatments by improving root architecture, maintaining photosystem II efficiency (ΦPSII), sustaining linear electron flow (LEF), and enhancing antioxidant enzyme activities, including superoxide dismutase (SOD), catalase (CAT), and peroxidase (POD). The specific objectives were to: (i) evaluate the effects of SA-based combinations on growth performance and root architecture of P. tuberosa under graded drought stress; and (ii) determine their influence on photosynthetic efficiency, chlorophyll fluorescence parameters, and antioxidant defense responses associated with drought tolerance. This research offers insight into the role of certain combinations of plant growth regulators (PGRs) that increase the drought tolerance of ornamental plants (tuberose) through alteration of physiological and biochemical processes. Results of this research will direct towards developing practical and sustainable methods of promoting floriculture in arid climates.

## Materials and methods

### Plant material and crop establishment

Tuberose (*Polianthes tuberosa* L.) plants were grown in 10 L plastic pots filled with a homogenized soil mixture consisting of sand (45%), silt (30%), clay (22%), and organic matter (3%), corresponding to a sandy loam texture. The experiment was conducted at the Cholistan Institute of Desert Studies, The Islamia University of Bahawalpur, Pakistan (29.35° N, 71.69° E) under semi-controlled greenhouse conditions (average day/night temperature 32 ± 3 °C/24 ± 2 °C; relative humidity 50–60%; 12 h photoperiod; PAR 600 ± 50 µmol m⁻² s⁻¹).

The experiment utilized a randomized complete block design (RCBD) with a factorial setup, consisting three different water regimes [90% (control), 70% (moderate drought), and 40% (severe drought) of soil water-holding capacity (WHC)] and five plant growth regulator (PGR) treatments, including control (no PGR), SA (300 ppm), SA + IAA (300 + 200 ppm), SA + AA (300 + 300 ppm), and SA + CS (300 + 300 ppm). The resulting 15 treatment combinations were replicated five times. Each replicate consisted of a single pot containing three plants, and the pot was considered the experimental unit for all statistical analyses. Thus, a total of 75 experimental units (15 treatment combinations × 5 replicates = 75) and 225 plants were included in the experiment. For variables measured on individual plants, data from the three plants within each pot were averaged prior to statistical analysis.

Healthy and uniform tuberose bulbs (average diameter 2.1 cm; fresh weight 8.0–8.5 g) were planted horizontally in each pot. PGR treatments were initiated at the three-leaf stage (BBCH scale 13). Water stress treatments were imposed based on pot water-holding capacity (WHC), including 90% (well-watered control), 70% (moderate drought stress), and 40% (severe drought stress). The 100% WHC was determined gravimetrically for each pot by saturating the soil with water until free drainage occurred, followed by 24 h drainage under standard laboratory conditions^27^, after which pot weight was recorded as the reference (100% WHC). Target moisture levels (90%, 70%, and 40% WHC) were maintained on a weight basis relative to this reference value. During the experiment, the pots were weighed at a consistent time twice each week. Water was added as necessary to keep the WHC levels stable. To ensure the accuracy of moisture levels, a digital soil tensiometer (LT1, 28 cm, Tensio-Technik, Geisenheim, Germany) was used after each weighing and watering session. These procedures were maintained for eight weeks straight, up until the first floret appeared. During the initial establishment phase, pots were irrigated twice per week, whereas irrigation frequency during later growth stages was adjusted to maintain the target WHC levels.

Plants were supplied with half-strength Hoagland nutrient solution according to Hoagland and Arnon^28^. The nutrient solution contained the following macronutrients and micronutrients (final concentrations in the nutrient solution): NH₄H₂PO₄, KNO₃, Ca(NO₃)₂, MgSO₄, H₃BO₃, MnCl₂·4 H₂O, ZnSO₄·7 H₂O, CuSO₄·5 H₂O, H₂MoO₄·H₂O, FeSO₄·7 H₂O chelated with EDTA. The nutrient solution was applied uniformly to all treatments throughout the experimental period. Half-strength Hoagland solution was used to avoid excessive nutrient availability and ensure uniform baseline nutrition under controlled pot conditions^29,30^.

### Application of plant growth regulators

Foliar applications were performed using a calibrated hand sprayer until uniform leaf wetness (point of incipient runoff) was achieved. Approximately 10 mL of solution was applied per pot at each application, ensuring consistent coverage of the foliage across all experimental units while avoiding excessive runoff at 25, 40, 55, and 70 days after planting and were selected to coincide with major developmental stages of tuberose, including active vegetative growth, rapid leaf expansion, floral stalk initiation, and pre-flowering development, thereby ensuring sustained physiological activity of the applied growth regulators throughout the experimental period.

The PGR stock solutions were prepared freshly before each application. Salicylic acid (SA; 300 ppm) was first dissolved in a small volume of ethanol (approximately 10% of final volume) under continuous magnetic stirring and then diluted with distilled water. Indole-3-acetic acid (IAA; 200 ppm) was prepared using the same procedure. Ascorbic acid (AA; 300 ppm) was directly dissolved in distilled water, whereas chitosan (CS; 300 ppm) (low molecular weight) was dissolved in 1% (v/v) acetic acid under constant stirring for 30 min until complete dissolution. To ensure the treatments remained stable and results were consistent, the pH of each solution was kept between 6.5 and 7.0 by adjusting with 0.1 M NaOH or HCl.

The PGR treatments included salicylic acid (SA), a combination of SA and indole-3-acetic acid (IAA), SA with ascorbic acid (AA), and SA with chitosan (CS). These combined treatments were freshly prepared by mixing the respective stock solutions just before each foliar application. The concentrations used were 300 ppm for SA, AA, and CS, while IAA was applied at 200 ppm. An untreated control treatment was included. The chosen concentrations and application schedule were based on prior research^31,32^. To enhance leaf wetting and absorption, Tween-20 (0.05%, v/v) was added to all spray solutions, including the control. Spraying was performed in the early morning under low light conditions to ensure consistent absorption. Furthermore, pots were rotated regularly during the experiment to reduce positional and environmental variations within the growth area.

### Growth and root morphological traits

At flowering, five plants per replication were randomly sampled. Roots were washed gently with deionized water. Recorded parameters included leaf area, shoot and root fresh and dry weights, stalk height, number of flowering stalks, and number of bulblets per plant.

Roots were separated at the hypocotyl junction and scanned using a flatbed root scanner (EPSON V800, J221B, Indonesia). Root morphological traits, including total length, surface area, volume, diameter, and tip number, were analyzed with WinRHIZO Pro 2021a (Regent Instruments, Canada). Leaf area was determined using a portable leaf area meter (AM350, ADC BioScientific, UK). Stalk height was measured from the base to the tip of the inflorescence with a graduated rod.

### Chlorophyll fluorescence and photosynthetic measurements

Chlorophyll fluorescence and photosynthetic parameters were measured 28 days after the initiation of water stress treatments using a portable MultispeQ fluorometer (PhotosynQ Inc., USA), following the protocol described by Kuhlgert et al. ^33^. Measurements were taken between 10:00 and 12:00 h on fully expanded fourth or fifth leaves from the apex. Prior to measurements, leaves were enclosed in aluminum clips for 30 min to ensure equilibration before measuring Fv/Fm.

Measured chlorophyll fluorescence parameters included maximum quantum efficiency of PSII (Fv/Fm), photochemical quenching (qP), fraction of open PSII centers (qL), and relative chlorophyll content. Photosynthetic performance was assessed by estimating linear electron flow (LEF), effective quantum yield of PSII (ΦPSII), and total non-photochemical quenching (NPQt).

### Antioxidant enzymes measurements

For antioxidant enzyme analysis, all biochemical parameters were expressed either on a fresh weight (FW) basis or protein basis depending on the nature of the assay, as per standard protocols to ensure methodological appropriateness and consistency. 0.5 g of fresh leaf tissue was homogenized in 50 mM potassium phosphate buffer (pH 7.8) containing 0.1 mM Na₂-EDTA, 0.3% (v/v) Triton X-100, and 1% (w/v) polyvinylpyrrolidone (PVP). The homogenate was centrifuged at 12,500 × g for 12 min at 4 °C, and the resulting supernatant was used as the crude enzyme extract.

Superoxide dismutase (SOD; EC 1.15.1.1) activity was determined by measuring the inhibition of nitroblue tetrazolium (NBT) photoreduction following the method of Giannopolitis and Ries^34^. Absorbance was recorded at 560 nm using a spectrophotometer (STA-4800, Stalwart Analytics, USA). One unit of SOD activity was defined as the amount of enzyme required to cause 50% inhibition of NBT photoreduction. Catalase (CAT; EC 1.11.1.6) and peroxidase (POD; EC 1.11.1.7) activities were assayed according to Liu et al. ^35^. CAT activity was determined by monitoring the decrease in absorbance at 240 nm due to H₂O₂ decomposition, whereas POD activity was quantified by measuring the increase in absorbance at 470 nm due to guaiacol oxidation. All enzyme activities were expressed on a protein basis (U mg⁻¹ protein) to ensure comparability across treatments, where protein content was determined from the same extract using the Bradford assay^36^.

### Total phenolic content and antioxidant capacity

Fresh leaves (5 g) were extracted in 20 mL of 90% methanol containing 2% HCl and 8% acetone. After centrifugation at 9000× g for 12 min, the supernatant was used for biochemical assays.

Total phenolics were quantified by the Folin–Ciocalteu method as described by Ainsworth and Gillespie^37^. After incubation for 75 min at room temperature, absorbance was measured at 765 nm using a spectrophotometer (STA-4800, Stalwart Analytics, USA). Total phenolics were quantified using gallic acid (3,4,5-trihydroxybenzoic acid) as an external standard and expressed as mg gallic acid equivalents (GAE) g⁻¹ fresh weight.

Total antioxidant capacity was assessed using the 2,2-diphenyl-1-picrylhydrazyl (DPPH) radical scavenging assay following the protocol of Mimica-Dukić et al. ^38^. A reaction mixture was prepared by mixing 150 µL of leaf extract with DPPH solution (0.004%, w/v). Samples were incubated in the dark for 30 min, and absorbance was measured at 517 nm. Antioxidant activity was expressed as percentage inhibition of DPPH radicals. To maintain alignment with standard methods for quantifying phytochemicals, all parameters related to metabolites, including total phenolics and antioxidant capacity, were reported based on fresh weight (FW).

### Assessment of total soluble protein

Total soluble proteins were determined by the Bradford assay^36^. One gram of fresh leaf tissue was homogenized in 10 mL ice-cold phosphate buffer (0.1 M, pH 7.0), centrifuged at 10,000 rpm for 15 min at 4 °C, and the supernatant was collected. For analysis, 50 µL of extract was mixed with 2.5 mL Bradford reagent, vortexed, incubated for 10 min at room temperature, and absorbance was measured at 595 nm. Protein concentration was quantified using a BSA standard curve (0–100 µg mL⁻¹) and expressed as mg g⁻¹ fresh weight. This protein quantification was also used for normalization of antioxidant enzyme activities (SOD, CAT, and POD) expressed as U mg⁻¹ protein.

### Relative water contents and electrolyte leakage

Leaf relative water contents were estimated according to the method of Masood et al. ^39^. Briefly, fresh leaves were cut 3 cm in length, weighed for fresh weight (FW) and soaked overnight. After this, the leaves were weighed for its turgid weight (TW), oven-dried at 65 ^◦^C (until the constants weight achieved) for its dry weight content (DW). The relative water contents were calculated by using following equation:

$${\rm RWC }(\%) =\:\frac{\boldsymbol{F}\boldsymbol{W}-\boldsymbol{D}\boldsymbol{W}\:}{\boldsymbol{T}\boldsymbol{W}-\boldsymbol{D}\boldsymbol{W}\:}\times\:100$$.

where FW = fresh weight, TW = turgid weight, and DW = dry weight.

Electrolyte leakage (EL) was measured according to the method of Dionisio-Sese and Tobita^40^. Ten leaf discs (15 mm) were placed in 30 mL deionized water and incubated at 25 °C for 2 h; the initial conductivity was recorded as EC₁. Samples were then autoclaved for 15 min at 100 °C, cooled, and the final conductivity measured as EC₂. The percentage of ion leakage was measured using the following formula:

$${\rm EL }=\:\frac{\boldsymbol{E}\boldsymbol{C}1}{\boldsymbol{E}\boldsymbol{C}2}\times\:100$$

### Statistical analysis

Data were analyzed using a two-way analysis of variance (ANOVA) to evaluate the effects of irrigation regime (drought stress), plant growth regulator (PGR) treatment, and their interaction. Statistical analyses were performed using Minitab v.16 (Minitab Inc., USA). Treatment means were compared using the least significant difference (LSD) test at *p* ≤ 0.05. Prior to ANOVA, assumptions of normality and homogeneity of variance were verified using the Shapiro–Wilk and Levene’s tests, respectively. Multivariate analyses were used alongside ANOVA to gain a more comprehensive understanding of the data. ANOVA provided evidence of treatment effects on separate traits independently, but the multivariate analysis revealed relationships of variables together as well as an overall pattern of what is being compared that cannot observed by analyzing at each trait individually. Principal component analysis (PCA), Pearson’s correlation analysis, and hierarchical cluster analysis (HCA) were performed using OriginPro 2024 (OriginLab, USA). PCA was conducted to simplify the overall data set while determining key variables influencing total variance across treatment groups. In addition, Pearson correlation analysis was conducted to assess the strength of association between traits as well as the direction of association between traits to demonstrate functional relationships between physiological and biochemical measures. Lastly, using hierarchical cluster analysis (HCA), variables that were similar to each other were clustered together to allow for classification of traits into a set of clusters that reflect how the measured traits respond together as a group. PCA was based on standardized data, and HCA was performed using the group average linkage method based on similarity coefficients to classify variables according to their association patterns. Pearson’s correlation coefficients were calculated to determine the strength and direction of relationships among variables. All graphical representations were generated using OriginPro 2024.

## Results

### Growth characteristics of *Polianthes tuberosa* L

**Fig. 1:**
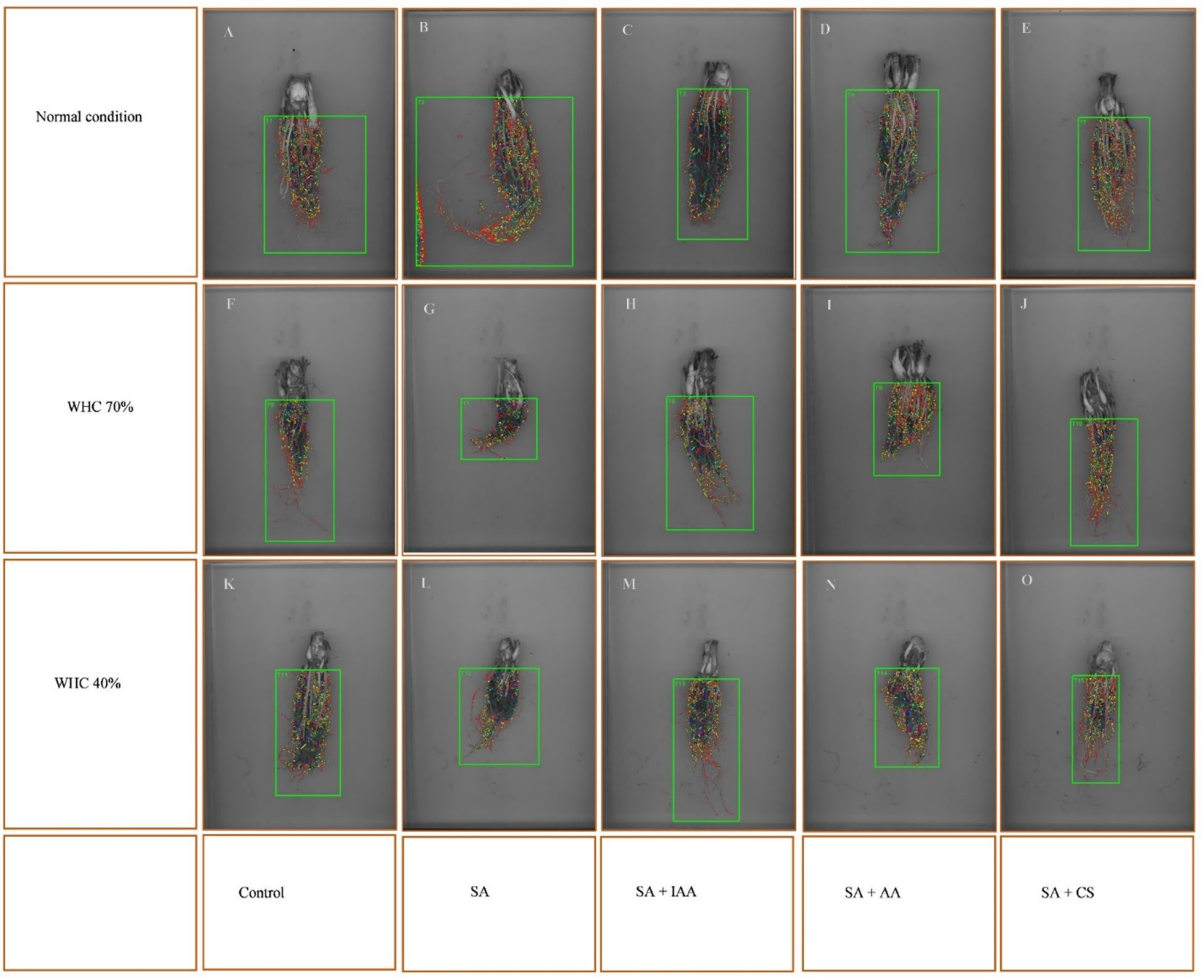
Digital scanning of plant root system under applied drought stress and PGRs in *Polianthes tuberosa* L. (SA; Salicylic Acid, IAA; Indole Acetic Acid, AA; Ascorbic Acid, CS; Chitosan).

Drought stress, plant growth regulators (PGRs), and their interaction significantly (*p* ≤ 0.05) affected the growth attributes of *Polianthes tuberosa* L. plants (Table 1; Fig. 1).

**Table 1:** Effect of drought stress and PGRs on the morphological attributes of Tuberose (***Polianthes tuberosa*** L. plants).

Plant growth regulators (PGRs)	Water Holding Capacity (WHC)							
	Normal Condition	WHC 70%	WHC 40%	Mean	Normal Condition	WHC 70%	WHC 40%	Mean
	**Leaf Area (cm** ^2^ **)**				**Shoot fresh weight (g)**			
Control	14.39 g	14.07 h	13.63I	**14.02D**	15.40 h	7.88 l	6.12 m	**9.84E**
Salicylic acid	15.48e	14.74f	13.31j	**14.52 C**	19.60f	15.00 h	9.20 k	**14.55D**
Salicylic acid + Indole Acetic Acid	23.58a	21.51b	18.49c	**21.09 A**	34.40a	23.20d	16.40 g	**24.55 A**
Salicylic acid + Ascorbic acid	14.69f	14.86f	12.76k	**14.07D**	25.00c	12.20j	14.00I	**17.33 C**
Salicylic acid + Chitosan	16.32d	15.65e	14.69f	**15.60B**	29.40b	21.40e	14.60hI	**21.44B**
Mean	**16.84 A**	**16.18B**	**14.56 C**		**24.73 A**	**15.80B**	**12.11 C**	
LSD@0.01	Interaction 32.87, Drought 14.71, PGRs 18.98				Interaction 1.33, Drought 0.59, PGRs 0.76			
	**Shoot dry weight (g)**				**Root fresh weight (g)**			
Control	6.08 h	3.04j	2.42k	**3.81E**	25.60e	15.50I	9.06k	**16.54E**
Salicylic acid	8.74e	6.18 h	3.90I	**6.30D**	33.80c	23.40f	16.00hi	**24.33 C**
Salicylic acid + Indole Acetic Acid	16.50a	11.16c	7.66f	**11.56 A**	43.80a	28.60d	20.40 g	**31.33 A**
Salicylic acid + Ascorbic acid	11.40c	4.06I	6.70 g	**7.44 C**	29.40d	15.24I	13.96j	**19.36D**
Salicylic acid + Chitosan	12.70b	9.30d	6.80 g	**9.55B**	37.80b	24.20f	17.00 h	**26.44B**
Mean	**11.12 A**	**6.68B**	**5.46 C**		**34.06 A**	**21.56B**	**15.18 C**	
LSD@0.01	Interaction 0.56, Drought 0.25, PGRs 0.32				Interaction 1.89, Drought 0.84, PGRs 1.09			
	**Root dry weight (g)**				**Stalk length (cm)**			
Control	12.80e	6.74 I	3.80j	**7.75E**	53.40c	33.60 g	26.20 h	**37.11 C**
Salicylic acid	16.17c	9.08 g	7.72 h	**10.96 C**	64.80b	45.20e	34.60 g	**48.00B**
Salicylic acid + Indole Acetic Acid	19.60a	13.88d	9.30 g	**14.32 A**	69.20a	52.00c	39.20f	**52.55 A**
Salicylic acid + Ascorbic acid	12.68e	6.84I	6.90I	**8.84D**	62.60b	48.00d	32.20 g	**48.00B**
Salicylic acid + Chitosan	17.06b	10.14f	8.02 h	**11.73 A**	64.00b	49.00d	37.80f	**49.22 A**
Mean	**15.68 A**	**9.40B**	**7.08 C**		**62.06 A**	**45.13B**	**33.73 C**	
LSD@0.01	Interaction 0.89, Drought 0.40, PGRs 0.51				Interaction 3.13, Drought 1.4, PGRs 1.8			
	**No. of florets stalk** ^-1^				**No of bulblets**			
Control	26.80 cd	26.40 cd	19.80e	**24.55 C**	15.00 h	8.38k	7.82k	**10.56E**
Salicylic acid	34.20b	27.60c	18.60e	**26.66 A**	23.40e	17.00 g	10.16j	**16.57 C**
Salicylic acid + Indole Acetic Acid	35.20ab	25.60d	15.60f	**25.66AB**	34.00a	25.92c	14.36 h	**24.93 A**
Salicylic acid + Ascorbic acid	36.40a	25.40d	18.80e	**26.44 A**	24.60d	12.08I	3.90 l	**13.72D**
Salicylic acid + Chitosan	35.60ab	26.20 cd	13.54 g	**25.18BC**	31.60b	19.40f	10.48j	**20.88B**
Mean	**33.73 A**	**26.26 B**	**17.11 C**		**26.00 A**	**16.60B**	**9.40 C**	
LSD@0.01	Interaction 1.83, Drought 0.81, PGRs1.05				Interaction 1.24, Drought 0.55, PGRs 0.71			

Data represents mean of five replicates. Different lowercase letters within a column and uppercase letters within a row indicate significant differences among treatments according to the least significant difference (LSD) test at *p* ≤ 0.01. WHC denotes water holding capacity. PGRs represent plant growth regulators. Interaction, drought, and PGR effects were tested using two-way ANOVA, and corresponding LSD values are presented at the bottom of each trait.

Drought stress significantly (*p* ≤ 0.05) reduced all growth parameters. Compared with normal conditions, severe drought (40% WHC) reduced leaf area from 16.84 to 14.56 cm² (13%), shoot fresh weight from 24.73 to 12.11 g (51%), shoot dry weight from 11.12 to 5.46 g (51%), root fresh weight from 34.06 to 15.18 g (55%), root dry weight from 15.68 to 7.08 g (54%), stalk length from 62.06 to 33.73 cm (45%), florets per stalk from 33.73 to 17.11 (49%), and bulblets per plant from 26.00 to 9.40 (64%). Moderate drought (70% WHC) also significantly (*p* ≤ 0.05) reduced all traits, including shoot fresh weight (36%), shoot dry weight (40%), root fresh weight (36%), root dry weight (40%), stalk length (27%), florets per stalk (22%), and bulblets per plant (36%) relative to non-stress conditions (Table 1).

PGR applications significantly influenced all growth attributes. Across treatments, SA + IAA recorded the highest mean values for leaf area (21.09 cm²), shoot fresh weight (24.55 g), shoot dry weight (11.56 g), root fresh weight (31.33 g), root dry weight (14.32 g), stalk length (52.55 cm), florets per stalk (25.66), and bulblets per plant (24.93), followed by SA + chitosan, while the lowest values were recorded in the untreated control (Table 1).

The drought × PGR interaction was significant (*p* ≤ 0.05), indicating differential responses of treatments across moisture regimes. Under normal conditions, SA + IAA produced the highest values for all growth parameters, including leaf area (23.58 cm²), shoot fresh weight (34.40 g), shoot dry weight (16.50 g), root fresh weight (43.80 g), root dry weight (19.60 g), stalk length (69.20 cm), florets per stalk (35.20), and bulblets per plant (34.00), while the control showed the lowest values. A similar trend was observed under 70% WHC and 40% WHC, where SA + IAA consistently maintained the highest growth performance across all measured traits, followed by SA + chitosan and SA + ascorbic acid, while the control remained lowest under both stress levels (Table 1).

**Fig. 2:**
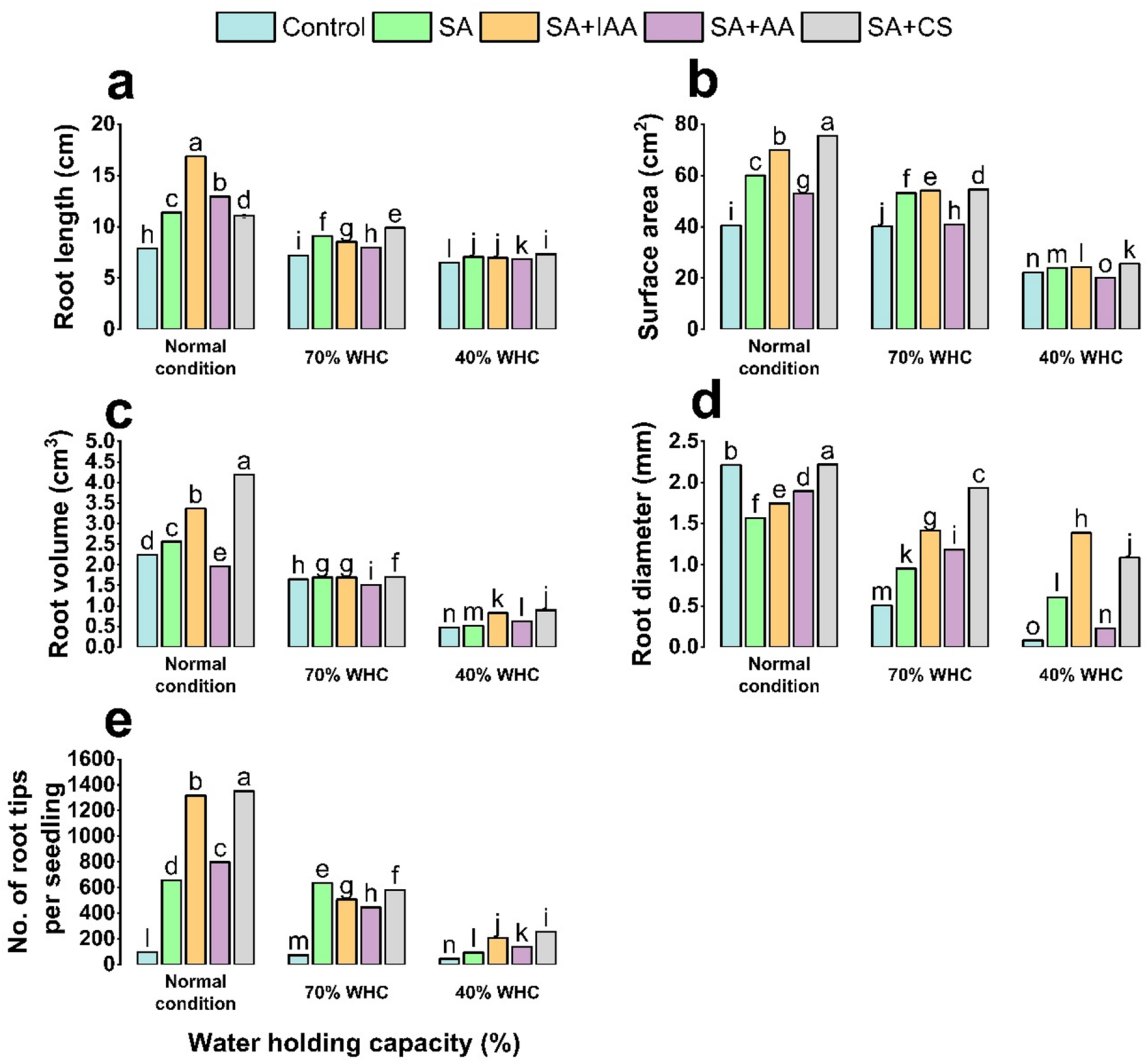
Effects of drought stress and plant growth regulators (PGRs) on root system attributes of *Polianthes tuberosa* L.: (a) root length, (b) root surface area, (c) root volume, (d) root diameter, and (e) number of root tips per seedling. Treatments included control, salicylic acid (SA), SA + indole-3-acetic acid (SA + IAA), SA + ascorbic acid (SA + AA), and SA + chitosan (SA + CS). Values are mean ± SE (*n* = 5). Different lowercase letters indicate significant differences among treatments according to the LSD test (*P* < 0.05).

Root morphological traits were significantly (*p* ≤ 0.05) affected by drought stress, PGR treatments, and their interaction (Fig. 2a–e). Overall, drought stress reduced all root traits, with more pronounced effects at 40% WHC than at 70% WHC. A significant drought stress × PGR interaction indicated that the response of root traits to PGR application depended on water availability.

Under normal conditions, PGR application substantially increased root growth compared to the control. Root length increased from 7.89 cm in the control to 11.07–16.87 cm in PGR-treated plants, with the highest values recorded in the SA + IAA treatment (16.87 cm). Root volume, surface area, and root tip number also showed strong increases, particularly under SA + IAA and SA + CS treatment. Root diameter showed comparatively limited variation among treatments, ranging from 1.56 to 2.22 mm (Fig. 2b-e).

Under 70% WHC, root traits declined; however, PGR application partially alleviated the effects of drought. Root length was higher in the SA + CS (9.91 cm) and SA (9.09 cm) groups than in the SA + IAA (8.54 cm), SA + AA (7.98 cm), and control (7.21 cm) groups. Root diameter and root surface area followed similar trends, with SA + CS generally showing the highest values for both.

Under severe drought (40% WHC), root growth was strongly inhibited, but PGRs improved plant performance relative to the control. Root length ranged from 6.49 cm (control) to 7.34 cm (SA + CS), while SA + IAA showed relatively higher root diameter (1.39 mm) and root tip number (208) than the other treatments.

Overall, the magnitude of PGR-induced improvements varied across drought levels, confirming a strong and consistent drought stress × PGR interaction for all root morphological traits.

### The effect on relative water contents and electrolyte leakage

Relative water content (RWC) and electrolyte leakage (EL) were significantly (*p* ≤ 0.05) affected by drought stress, PGR application, and their interaction (Table 2). The main effect of drought stress showed a significant (*p* ≤ 0.05) decline in RWC with increasing water deficit, decreasing from 81.48% under normal conditions to 78.20% at 70% WHC and 72.40% at 40% WHC, corresponding to reductions of 4% and 11%, respectively (Table 2). In contrast, EL increased significantly with drought severity, rising from 30.31% under normal conditions to 34.20% and 36.12% under 70% and 40% WHC, respectively (Table 2).

**Table 2:** Effect of drought stress and PGRs on physiological and antioxidant attributes of tuberose (***Polianthes tuberosa L***. plants).

Plant growth regulators	Water Holding Capacity (WHC)							
	Normal Condition	WHC 70%	WHC 40%	Mean	Normal Condition	WHC 70%	WHC 40%	Mean
	**Relative Water Content (%)**				**Total Phenolics (mg GAE/100 g FW)**			
Control	76.29 h	69.47 k	65.92 m	**70.56 D**	3604.19d	3201.81j	3055.62n	**3287.20D**
Salicylic acid	78.96 e	76.88 g	67.86 l	**74.57 C**	3703.24b	3243.24 g	3087.52 m	**3344.70B**
Salicylic acid + Indole Acetic Acid	82.14 c	81.37 d	78.19 f	**80.57 B**	3674.19c	3209.90i	3102.28 l	**3328.80 C**
Salicylic acid + Ascorbic acid	77.18 g	75.36 i	71.53 j	**74.69 C**	3716.09a	3273.72f	3128.47k	**3372.80 A**
Salicylic acid + Chitosan	92.85 a	87.92 b	78.54 ef	**86.43 A**	3518.00e	3230.85 h	2887.04o	**3212.00E**
Mean	81.48 A	78.20 B	72.40 C		**3643.10 A**	**3231.90B**	**3052.20 C**	
LSD@0.05	Interaction 0.46, drought 0.21, PGRs 0.27				Interaction 6.51, Drought 2.91, PGRs 3.76			
	**Electrolyte Leakage (%)**				**Total Soluble Proteins (mg/g FW)**			
Control	38.72 b	40.90 a	41.37 a	**40.33 A**	15.25c	11.08 g	18.84ab	**15.06 A**
Salicylic acid	31.35 ef	34.82 d	36.84 c	**34.33 B**	12.22f	11.26 g	18.32b±	**13.94 C**
Salicylic acid + Indole Acetic Acid	27.91 g	31.42 e	34.48 d	**31.27 C**	14.45d	11.19 g	18.49ab	**14.71B**
Salicylic acid + Ascorbic acid	31.92 e	34.37 d	36.36 c	**34.21 B**	13.28e	11.11 g	18.92a±	**14.43B**
Salicylic acid + Chitosan	21.68 h	30.62 f	31.57 e	**27.95 D**	11.59 g	11.06 g	18.66ab	**13.77 C**
Mean	**30.31 C**	**34.2 B**	**36.12 A**		**13.36B**	**11.14 C**	**18.66 A**	
LSD@0.05	Interaction 0.69, drought 0.31, PGRs 0.39				Interaction 0.56, Drought 0.25, PGRs 0.32			

Data represents mean of five replicates. Different lowercase letters within a column and uppercase letters within a row indicate significant differences among treatments according to the least significant difference (LSD) test at *p* ≤ 0.01. WHC denotes water holding capacity. PGRs represent plant growth regulators. Interaction, drought, and PGR effects were tested using two-way ANOVA, and corresponding LSD values are presented at the bottom of each trait.

Among PGR treatments, SA + chitosan recorded the highest mean RWC (86.43%), followed by SA + IAA (80.57%), SA + ascorbic acid (74.69%), SA alone (74.57%), and the control (70.56%). For EL, SA + chitosan showed the lowest mean value (27.95%), followed by SA + IAA (31.27%), SA + ascorbic acid (34.21%), SA alone (34.33%), and the control (40.33%) (Table 2).

The interaction between drought stress and PGR application was significant (*p* ≤ 0.05) for RWC. Under normal conditions, RWC ranged from 76.29% to 92.85%, with SA + chitosan showing the highest value (92.85%), followed by SA + IAA (82.14%), SA + ascorbic acid (77.18%), SA alone (78.96%), and control (76.29%). Under 70% WHC, RWC ranged from 69.47% to 87.92%, with SA + chitosan again showing the highest value (87.92%), followed by SA + IAA (81.37%), SA + ascorbic acid (75.36%), SA alone (76.88%), and control (69.47%). Under 40% WHC, RWC ranged from 65.92% to 78.54%, with SA + chitosan (78.54%) and SA + IAA (78.19%) showing the highest values, followed by SA + ascorbic acid (71.53%), SA alone (67.86%), and control (65.92%).

For EL the interaction effect shows that under normal conditions, values ranged from 21.68% to 38.72%, with SA + chitosan showing the lowest EL (21.68%), followed by SA + IAA (27.91%), SA + ascorbic acid (31.92%), SA alone (31.35%), and control (38.72%). Under 70% WHC, EL ranged from 30.62% to 40.90%, with SA + chitosan showing the lowest value (30.62%), followed by SA + IAA (31.42%), SA + ascorbic acid (34.37%), SA alone (34.82%), and control (40.90%). Under 40% WHC, EL ranged from 31.57% to 41.37%, with SA + chitosan (31.57%) and SA + IAA (34.48%) showing the lowest values, followed by SA + ascorbic acid (36.36%), SA alone (36.84%), and control (41.37%).

### Effect on total phenolic and protein contents

Total phenolic content (TPC) was significantly (*p* ≤ 0.05) affected by drought stress and plant growth regulator (PGR) application (Table 2). The main effect of drought stress showed a significant decline in TPC with increasing water deficit, decreasing from 3643.10 mg GAE/100 g FW under normal conditions to 3231.90 mg GAE/100 g FW at 70% WHC and 3052.20 mg GAE/100 g FW at 40% WHC, corresponding to reductions of 11% and 16%, respectively, compared to normal conditions.

Among PGR treatments, SA + ascorbic acid recorded the highest mean TPC (3372.80 mg GAE/100 g FW), followed by SA alone (3344.70 mg GAE/100 g FW) and SA + IAA (3328.80 mg GAE/100 g FW), while SA + chitosan showed the lowest value (3212.00 mg GAE/100 g FW) (Table 2).

The interaction between drought stress and PGR application significantly (*p* ≤ 0.05) affected TPC (Table 2). Under normal conditions, TPC ranged from 3518.00 to 3716.09 mg GAE/100 g FW. The highest value was recorded in SA + ascorbic acid (3716.09 mg GAE/100 g FW), followed by SA alone (3703.24 mg GAE/100 g FW), SA + IAA (3674.19 mg GAE/100 g FW), control (3604.19 mg GAE/100 g FW), and SA + chitosan (3518.00 mg GAE/100 g FW).

Under moderate drought (70% WHC), TPC decreased across all treatments. Values ranged from 3201.81 to 3273.72 mg GAE/100 g FW. The highest TPC was observed in SA + ascorbic acid (3273.72 mg GAE/100 g FW), followed by SA + chitosan (3230.85 mg GAE/100 g FW), SA alone (3243.24 mg GAE/100 g FW), SA + IAA (3209.90 mg GAE/100 g FW), and control (3201.81 mg GAE/100 g FW).

Under severe drought (40% WHC), TPC ranged from 2887.04 to 3128.47 mg GAE/100 g FW. The highest TPC was recorded in SA + ascorbic acid (3128.47 mg GAE/100 g FW), followed by SA + IAA (3102.28 mg GAE/100 g FW), SA alone (3087.52 mg GAE/100 g FW), control (3055.62 mg GAE/100 g FW), and SA + chitosan (2887.04 mg GAE/100 g FW).

Under drought main effects, total soluble protein (TSP) content decreased significantly (*p* ≤ 0.05) from 13.36 mg g⁻¹ FW under normal conditions to 11.14 mg g⁻¹ FW at 70% WHC, followed by an increase to 18.66 mg g⁻¹ FW at 40% WHC. Among PGR treatments, the highest mean TSP was recorded in the control (15.06 mg g⁻¹ FW), followed by SA + IAA (14.71 mg g⁻¹ FW), SA + ascorbic acid (14.43 mg g⁻¹ FW), and SA + chitosan (13.77 mg g⁻¹ FW) (Table 2).

Under moderate drought (70% WHC), TSP values ranged from 11.06 to 11.26 mg g⁻¹ FW. The highest value was recorded in SA (11.26 mg g⁻¹ FW), followed by SA + IAA (11.19 mg g⁻¹ FW), SA + ascorbic acid (11.11 mg g⁻¹ FW), SA + chitosan (11.06 mg g⁻¹ FW), and control (11.08 mg g⁻¹ FW) (Table 2).

Under severe drought (40% WHC), TSP increased markedly across all treatments, ranging from 18.32 to 18.92 mg g⁻¹ FW. The highest TSP was recorded in SA + ascorbic acid (18.92 mg g⁻¹ FW), followed by SA + chitosan (18.66 mg g⁻¹ FW), SA + IAA (18.49 mg g⁻¹ FW), control (18.84 mg g⁻¹ FW), and SA (18.32 mg g⁻¹ FW) (Table 2).

The interaction between drought stress and PGR application significantly (*p* ≤ 0.05) affected TSP (Table 2). Under normal conditions, TSP ranged from 11.59 to 15.25 mg g⁻¹ FW, with the highest value recorded in the control (15.25 mg g⁻¹ FW), followed by SA + IAA (14.45 mg g⁻¹ FW), SA + ascorbic acid (13.28 mg g⁻¹ FW), SA (12.22 mg g⁻¹ FW), and SA + chitosan (11.59 mg g⁻¹ FW) (Table 2).

### The effect on chlorophyll and photosynthetic attributes

**Fig. 3:**
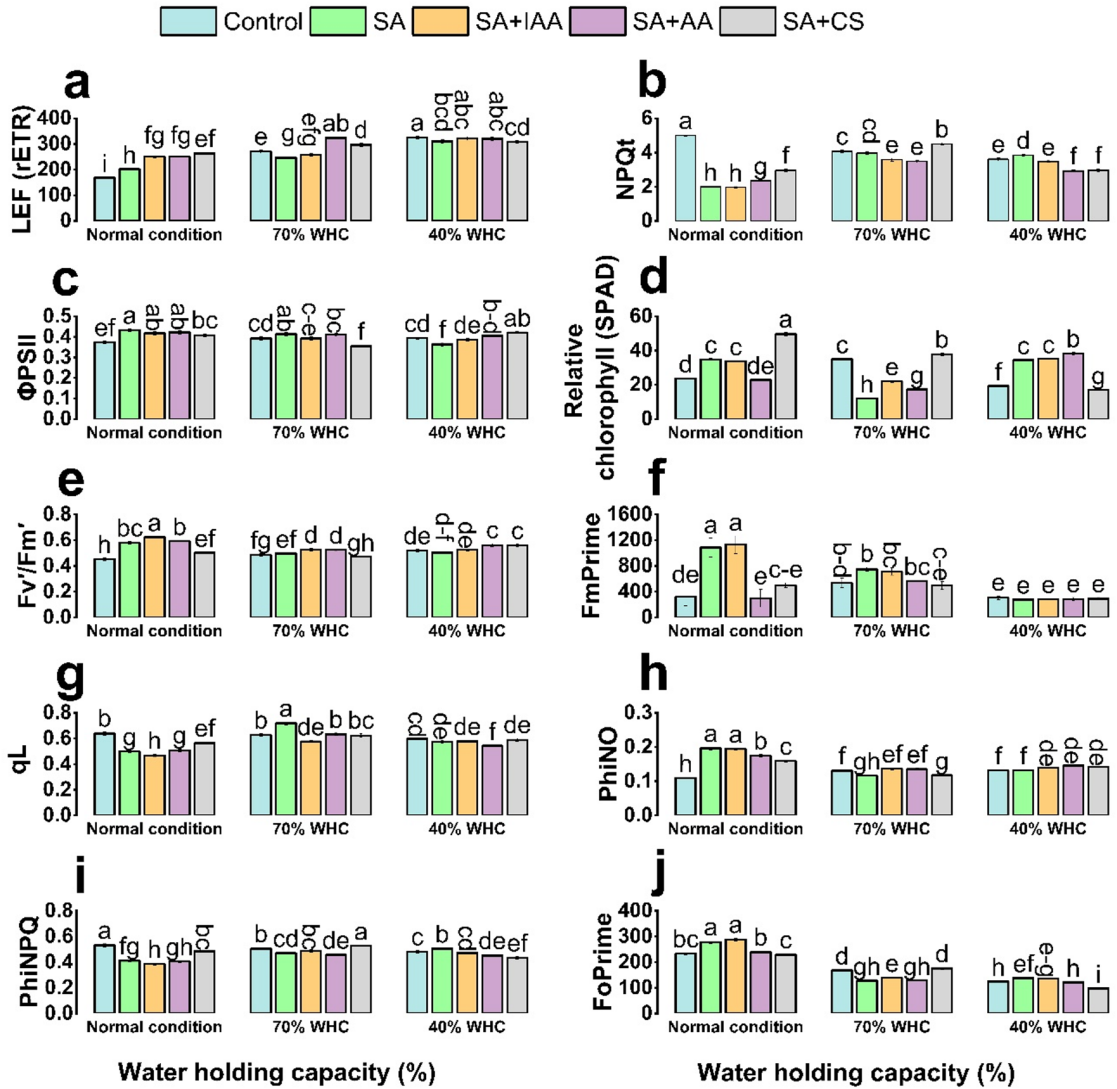
Effects of drought stress and plant growth regulators (PGRs) on photosynthetic performance of *Polianthes tuberosa* L. under different water-holding capacities (WHC). (a) Linear electron flow [LEF (rETR)], (b) total non-photochemical quenching (NPQt), (c) PSII operating efficiency (ΦPSII), (d) relative chlorophyll (SPAD), (e) maximum quantum efficiency of PSII in the light-adapted state (Fv′/Fm′), (f) maximum fluorescence in the light-adapted state (Fm′), (g) coefficient of photochemical quenching (qL), (h) quantum yield of non-regulated energy dissipation (ΦNO), (i) quantum yield of regulated energy dissipation (ΦNPQ), and (j) minimum fluorescence in the light-adapted state (Fo′) measured under normal (100% WHC), moderate (70% WHC), and severe (40% WHC) drought conditions. Treatments included control, salicylic acid (SA), SA + indole-3-acetic acid (SA + IAA), SA + ascorbic acid (SA + AA), and SA + chitosan (SA + CS). Values are mean ± SE (*n* = 5). Different lowercase letters indicate significant differences among treatments (LSD test, *P* < 0.05).

Photosynthetic performance and chlorophyll-related parameters were significantly (*p* ≤ 0.05) affected by drought stress, PGR application, and their interaction (Fig. 3a-j).

Under normal conditions, PGR application significantly improved linear electron flow (LEF) compared with the control (168.2), with the highest values recorded in SA + CS (263.0) and SA + AA (251.3), followed by SA + IAA (250.0). Total non-photochemical quenching (NPQt) was strongly reduced in SA + IAA (1.97) and SA + AA (2.36) compared with the control (5.01), indicating significant differences in energy dissipation regulation under non-stress conditions. Similarly, PSII operating efficiency (ΦPSII) increased in SA + IAA (0.418) and SA + AA (0.423) compared with the control (0.373), while relative chlorophyll content (SPAD values) was highest in SA + CS (49.60) compared with the control (23.72). Maximum quantum efficiency of PSII (Fv′/Fm′) was also improved in SA + IAA (0.622) and SA + AA (0.592) relative to untreated plants.

Under moderate drought (70% WHC), LEF increased in all PGR treatments compared with the control (270.46), with the highest value observed in SA + AA (323.71), followed by SA + CS (296.43). ΦPSII and photochemical quenching (qL) were higher in SA + IAA (0.392 and 0.576) and SA + AA (0.411 and 0.542) compared with untreated plants. NPQt showed treatment-dependent variation, ranging from 3.48 (SA + AA) to 4.50 (SA + CS), compared with 4.07 in the control, confirming a significant drought × PGR interaction in energy dissipation responses. Relative chlorophyll content decreased in SA + AA (17.25) compared with control (34.93), while SA + CS showed higher chlorophyll content (37.78), indicating contrasting PGR effects under moderate stress (Fig. 3).

Under severe drought (40% WHC), photosynthetic efficiency declined significantly in untreated plants, as reflected by reduced ΦPSII (0.394) and Fv′/Fm′ (0.518). However, SA + AA (0.405) and SA + IAA (0.386) maintained relatively higher ΦPSII values. qL was also higher in SA + AA (0.542) and SA + CS (0.588) compared with control (0.597). Non-regulated energy dissipation (ΦNO) increased in untreated plants, while ΦNPQ was higher in PGR-treated plants, particularly SA + IAA (0.471) and SA + AA (0.451), indicating improved regulated energy dissipation under severe stress conditions.

Overall, the significant interaction between drought and PGRs demonstrates that the magnitude of PGR-induced improvements depended strongly on WHC level. SA + IAA and SA + AA consistently maintained higher photochemical efficiency and more balanced energy partitioning under moderate and severe drought compared with the control, confirming their role in improving drought resilience.

### The effect on antioxidant enzymes activities

**Fig. 4:**
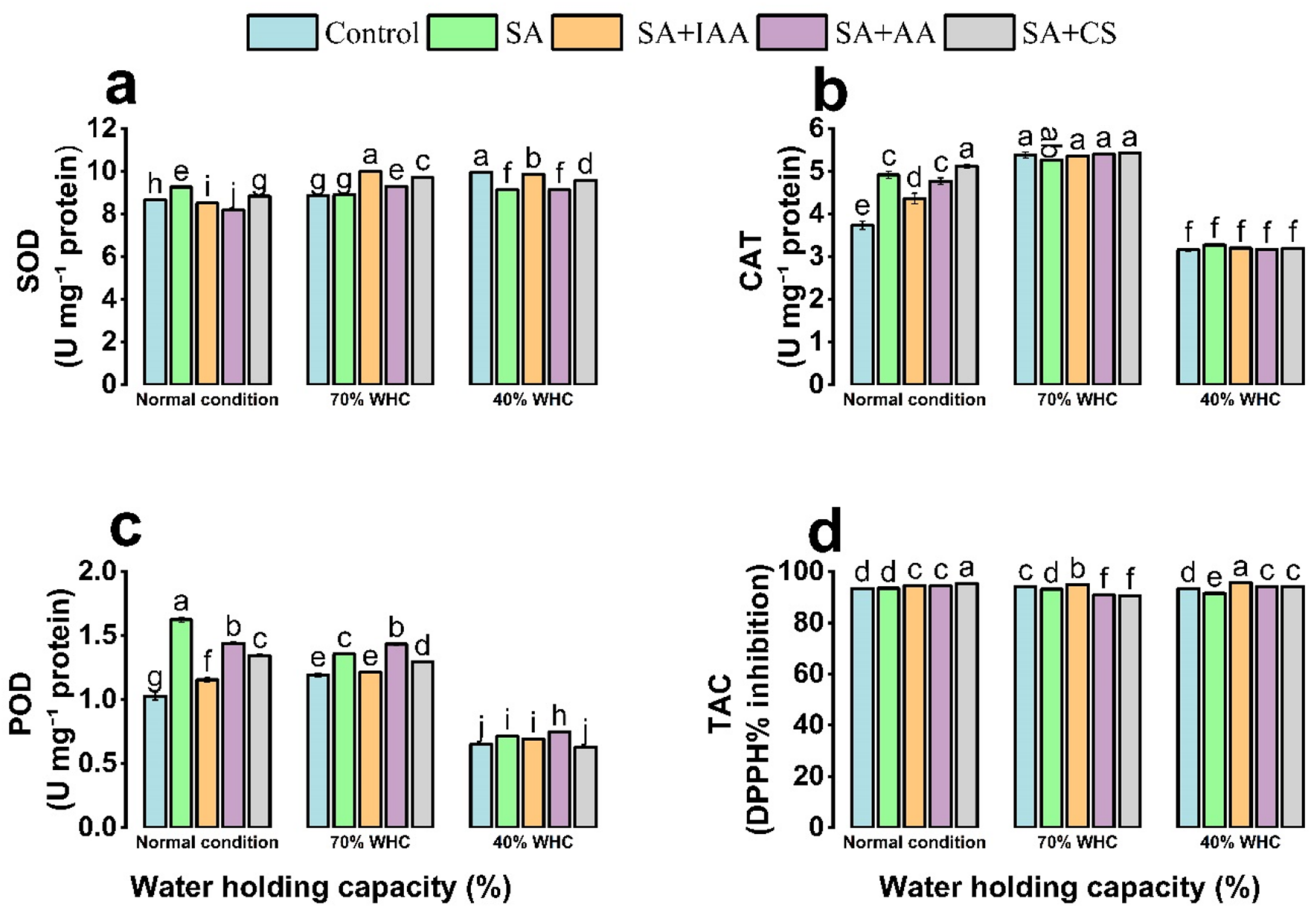
Effect of drought stress and plant growth regulators (PGRs) on antioxidant defense and total antioxidant capacity of *Polianthes tuberosa* L. plants under different water-holding capacities (WHC). (a) Superoxide dismutase (SOD), (b) catalase (CAT), (c) peroxidase (POD), and (d) total antioxidant capacity measured as DPPH radical scavenging activity (TAC). Plants were grown under normal conditions (90% WHC), moderate drought stress (70% WHC), and severe drought stress (40% WHC) and treated with salicylic acid (SA), salicylic acid + indole-3-acetic acid (SA + IAA), salicylic acid + ascorbic acid (SA + AA), and salicylic acid + chitosan (SA + CS), along with an untreated control. Values represent mean ± SE (*n* = 5). Different lowercase letters above bars indicate significant differences among treatments according to LSD test at *P* < 0.05.

Foliar application of PGRs enhanced the activities of key antioxidant enzymes, including superoxide dismutase (SOD), peroxidases (POD), catalase (CAT), and total antioxidant capacity, under both normal and stress conditions (Fig. 4). SA alone increased SOD by 7.05% (Fig. 4a), CAT by 31.70% (Fig. 4b), and POD by 57.74% (Fig. 4c) compared to the control, while SA + CS maximally improved total antioxidant activity by 2.16% (Fig. 4d).

Drought stress significantly affected enzyme activities (*p* ≤ 0.05). Moderate drought, relative to control, increased SOD by 2.43%, CAT by 44.28%, POD by 15.72%, and total antioxidant activity by 0.71%, whereas severe drought reduced CAT (15.22%), POD (36.80%), and total antioxidant activity (0.05%), with SOD remaining relatively stable (Fig. 4).

The interaction effect shows that foliar application of PGRs mitigated the adverse effects of drought and further enhanced enzymatic activities in stressed plants. Under moderate drought, SA + IAA improved SOD (12.87%) and total antioxidant activity (1.96%), while SA + AA enhanced POD under both moderate (20.50%) and severe (15.23%) drought. CAT activity was less responsive. Overall, SA-based PGR combinations consistently elevated antioxidant enzyme activities across conditions.

### Multivariate analysis

**Fig. 5:**
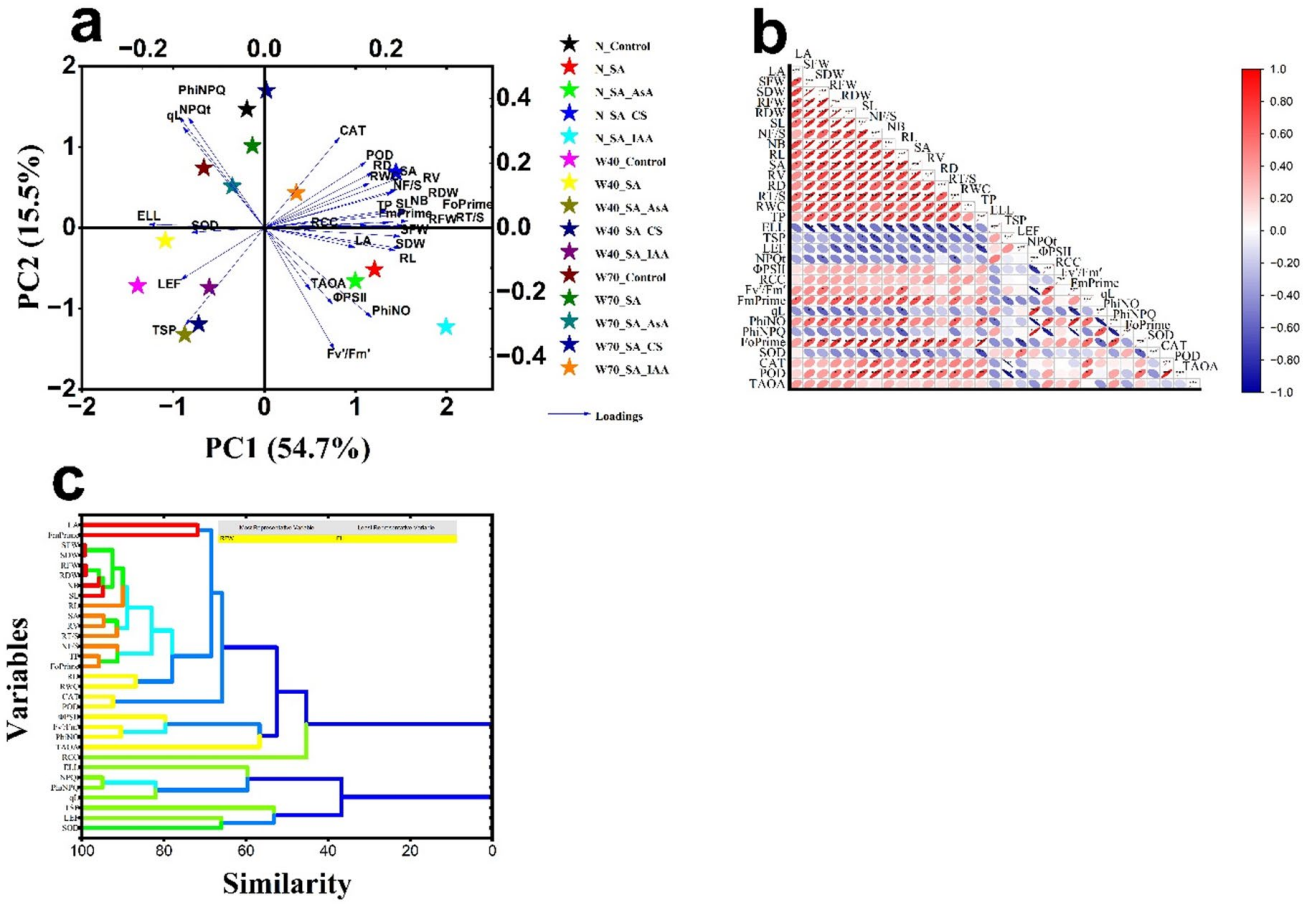
Multivariate analysis of growth, chlorophyll fluorescence, physiological, and biochemical traits of *Polianthes tuberosa* L. under different water stress levels and foliar application of plant growth regulator (PGRs) treatments. (a) Principal component analysis (PCA) biplot showing the distribution of treatment combinations (Water stress levels × PGR) and variable loadings; (b) Pearson’s correlation matrix illustrating the strength and direction of associations among measured variables; red and blue colors indicate positive and negative correlations, respectively, and color intensity corresponds to correlation magnitude. * p < = 0.05 ** p < = 0.01 *** p < = 0.001 and (c) Hierarchical cluster analysis (HCA) dendrogram depicting similarity relationships among variables based on Euclidean distance. Treatments consisted of three irrigation regimes based on water holding capacity (WHC): normal irrigation (90% WHC), moderate water stress (70% WHC), and severe water stress (40% WHC), combined with five PGR treatments: control (no PGR), salicylic acid (SA), salicylic acid + indole-3-acetic acid (SA + IAA), salicylic acid + ascorbic acid (SA + AsA), and salicylic acid + chitosan (SA + CS). Fo′, minimum fluorescence in light; Fm′, maximum fluorescence in light; Fv′/Fm′, maximum efficiency of PSII in light; ΦPSII, effective quantum yield of PSII; ΦNPQ, regulated non-photochemical energy dissipation; ΦNO, non-regulated energy dissipation; NPQt, total non-photochemical quenching; qL, coefficient of photochemical quenching; CAT, catalase; POD, peroxidase; SOD, superoxide dismutase; TAOA, total antioxidant activity; RWC, relative water content; TP, total phenolics; SFW, shoot fresh weight; SDW, shoot dry weight; RDW, root dry weight; RL, root length; LA, leaf area; ELL, electrolyte leakage.

Principal component analysis (PCA) (Fig. 5a) revealed that the first two principal components (PC1 and PC2) explained 70.2% of the total variance (PC1 = 54.7%; PC2 = 15.5%). PC1 was positively associated with growth and biomass-related traits, including shoot fresh weight (SFW), shoot dry weight (SDW), root dry weight (RDW), root length (RL), total phenolics (TP), relative water content (RWC), root volume (RV), and chlorophyll fluorescence parameters such as Fo′, Fm′, and ΦPSII. Antioxidant enzymes including catalase (CAT) and peroxidase (POD) also showed strong positive loadings along PC1, indicating their contribution to improved plant performance under favorable or PGR-amended conditions. In contrast, PC1 showed negative associations with non-photochemical quenching parameters (NPQt, ΦNPQ), excitation pressure (qL), electrolyte leakage (ELL), and superoxide dismutase (SOD), which were mainly associated with stress-induced responses.

PC2 separated treatments primarily based on photochemical efficiency and stress dissipation mechanisms. Parameters such as Fv′/Fm′, ΦNO, ΦPSII, and total antioxidant activity (TAOA) contributed strongly to the negative side of PC2, whereas ΦNPQ, NPQt, and qL loaded positively, reflecting differential energy partitioning within PSII under varying water stress levels.

The PCA score plot showed distinct clusters of the treatments. Well-watered and 70% WHC treatments with SA-based PGRs (SA + IAA, SA + AsA, SA + CS) were grouped on the positive PC1 side, exhibited increased growth, photosynthesis, and antioxidant activity, whereas 40% WHC treatments, especially the controls and single-PGR treatments, were grouped on the negative PC1/PC2 side, which indicated stress-induced inhibition (Fig. 5a). The PCA results were further confirmed by correlation analysis (Fig. 5b), showing strong positive correlations between biomass traits (LA, SFW, SDW, RDW, RL, RV, and NF/S), while RWC and ΦPSII showed significant positive correlation. The growth parameters and the fluorescence parameters were found to be positively correlated with CAT and POD. On the other hand, there were positive correlations among ELL, NPQt, ΦNPQ and qL, while negative associations between the biomass and the photochemical traits were observed (Fig. 5b).

The Hierarchical cluster analysis (HCA) (Fig. 5c) showed that there were two main clusters. The first cluster included growth-related traits along with RWC and Fo′, which showed high similarity between morphological and water-status parameters. The second cluster included chlorophyll fluorescence and biochemical attributes of the plant leaves. Amongst these, Fv′/Fm′, TAOA, CAT, and POD were strongly correlated and grouped into a close cluster, while ELL, NPQt, ΦNPQ, and qL formed a close cluster of stress-related variables. RDW had a central location in the growth cluster, while ELL had a more scattered growth cluster (Fig. 5c).

In general, the multivariate analyses clearly showed the irrigation treatment and PGR treatment effects, demonstrating a coordinated variation between growth, water relations, fluorescence characteristics, and antioxidant responses.

## Discussion

In this study drought stress caused a significant decrease in growth, biomass production, and quality of flowers of *Polianthes tuberosa* L., consistent with previous reports in ornamental and field crops^41^. This observed growth inhibition is mainly due to reduction in leaf water potential, stomatal closure and CO₂ assimilation and consequently the availability of photosynthate and cell expansion^42^.

The present study shows that reduction in leaf area, plant height and number of flower producing stems under drought stress were closely correlated with significant reduction in relative water content (RWC), chlorophyll concentration and net photosynthesis (Fig. 3). It was observed that the growth inhibition of tuberose was directly associated with reduction in water status and carbon assimilation and not only due to morphological limitations. These findings agreed with earlier studies that have shown that drought had a negative impact on flowering stem growth and, therefore on cut flower quality in tuberose^2,43^. Similarly, Ali et al. ^4^, reported that drought significantly reduced flower abortion, number of florets per stalk, bulb yield, and flowering spike length in tuberose. Such morphological impacts under drought stress have direct implications for marketability and profitability in floriculture.

Under drought conditions, the endogenous hormonal balance is substantially altered, with ABA acting as the central stress signal that coordinates stomatal closure, and growth regulation. In this context, exogenous salicylic acid (SA) functions as a critical hormonal modulator capable of fine-tuning stress responses and improving physiological stability under drought. Although ABA levels were not quantified in this study, the improved physiological performance observed following SA application suggests a potential interaction with drought-responsive signaling pathways. Growing evidence suggests that SA modulates guard cell ion transport, Ca²⁺ signaling, and ROS-mediated pathways, which may contribute to improved stomatal regulation and maintenance of photosynthetic activity under stress conditions. ^44^.

However, in the current study, drought-induced growth inhibition in tuberose plants was significantly alleviated by the exogenous application of PGRs, particularly salicylic acid (SA) combined with indole acetic acid (IAA). PGRs are known to enhance plant growth by modulating physiology and biochemical processes under both normal and drought-stressed conditions. This improvement may be attributed to hormonal adjustments improved antioxidant regulation, and enhanced photosynthetic stability^4,45,46^. SA has been shown to enhance root architecture and promote nutrient uptake from soil^47^. Additionally, SA contributes to osmoprotectant accumulation and activation of antioxidant enzymes, which protect plants from abiotic stress^48^. The combined application of SA and IAA likely provides a synergistic effect, where SA enhances stress tolerance while IAA sustains growth processes under reduced oxidative damage. This interpretation is supported by the higher ΦPSII, LEF, and chlorophyll content observed in these treatments (Fig. 3), indicating improved photochemical efficiency under drought conditions. The physiological responses observed in the present study are consistent with the evidence from the drought studies on fennel which showed that antioxidant defense and stability of photosynthesis are coordinated by SA ^49^.

The limited growth of *P. tuberosa* under extreme drought (WHC 40%) may also be attributed to a reduction in relative water content (RWC), leaf chlorophyll and LEF and ΦPSII downregulation (Fig. 3). The stomatal closure due to reduced RWC reduces the availability of CO₂ in leaves and decreases photosynthetic efficiency^50^. Under low CO₂, Rubisco activity is reduced, which impacts the Calvin cycle performance^51^. Additionally, the impaired activity of photosystem II results in an overproduction of reactive oxygen species (ROS), resulting in lipid peroxidation and damage to the membranes^52^. Low RWC and high electrolyte leakage suggest that oxidative stress was a direct contributor to decreased photochemical efficiency. In moderate drought (70% WHC) stress, photosynthetic efficiency parameters (ΦPSII and Fv′/Fm′) remained relatively unchanged when compared to severe drought stress, indicating partial acclimation of the plant to moderate drought stress in tuberose. The stability in this case probably was achieved by the action of SA which maintained the redox state and protects the function of PSII and thereby reduced the non-regulated energy dissipation (NPQt, ΦNO) as presented in Fig. 3.

Under drought stress, plants activate antioxidant defense systems to mitigate excessive ROS production. ROS, including superoxide (O₂•⁻), hydrogen peroxide (H₂O₂), hydroxyl radicals (•OH), and singlet oxygen, can damage lipids, proteins, and nucleic acids, causing cellular malfunction^53^. The balance between ROS generation and scavenging determines whether ROS act as damaging agents or signaling molecules. Plants employ both enzymatic antioxidants (SOD, CAT, POD, APX) and non-enzymatic antioxidants (ascorbic acid, glutathione, carotenoids, flavonoids) to detoxify ROS^54^. The present study showed that drought stress significantly increased the activities of SOD, POD, CAT, and overall antioxidant capacity, although activity declined at severe drought levels except for SOD (Fig. 4), consistent with previous reports in *P. tuberosa*^55^. SA-treated plants exhibited significantly higher activities of SOD, CAT, and POD under drought (Fig. 4). This enhanced antioxidant capacity was associated with lower electrolyte leakage and improved PSII performance, demonstrating that SA mediated drought tolerance in tuberose was closely tied to improved redox homeostasis and photochemical stability^56^. Among phytohormones, SA plays a key role as a defense elicitor, regulating antioxidant enzymes and secondary metabolite pathways^57^. In this study, SA alone or in combination with other PGRs effectively enhanced drought tolerance. SA also influences seed germination, vegetative growth, photosynthesis, flower bud induction, and flower quality by inhibiting ethylene and ABA pathways, thereby improving flower stalk longevity^58^. Additionally, recent studies have reported that combined biostimulant applications enhance drought tolerance through coordinated physiological and biochemical adjustments^59,60^, further supporting the synergistic effects observed in the present study. These findings are consistent with previous studies showing synergistic effects of SA and chitosan in enhancing antioxidant activity in *Achillea gypsicola*^61^ and the combination of SA and ascorbic acid in improving SOD and CAT activity under heat stress in wheat^62^. Furthermore, multivariate analysis showed that SA-based PGR combinations effectively reprogram drought responses, shifting plants from a stress-dominated state toward a more balanced, growth-supportive physiology, consistent with recent SA-mediated regulation studies^63,64^. PC1 (54.7% variance) defined a clear performance–stress gradient, where growth, water status, PSII efficiency, and antioxidant activity were positively associated, while membrane damage and energy dissipation showed opposing trends, confirming drought-induced disruption of membrane stability and photosynthetic energy balance^50^.

SA + IAA, SA + AsA, and SA + CS treatments clustered with well-watered and 70% WHC conditions, indicating partial recovery of physiological function. This aligns with evidence that SA and chitosan enhance PSII performance, reduce lipid peroxidation, and strengthen antioxidant defenses under drought^64^. Correlation and clustering analyses further separated stress markers, reinforcing the tight coupling between biomass maintenance, photosynthetic protection, and reduced energy loss. Overall, SA-based combinations improved drought tolerance through coordinated regulation of water relations, antioxidant metabolism, and PSII energy partitioning, consistent with reported synergistic effects of biostimulants under abiotic stress^65^.

## Conclusions

The combined foliar application of salicylic acid (SA) and different growth regulators (indole acetic acid (IAA), chitosan, and ascorbic acid) improved drought-related responses in *Polianthes tuberosa* L. under controlled pot experiment conditions by influencing growth, physiological, and biochemical responses traits. However, the responses were trait-dependent across treatments. SA + IAA was comparatively more effective in enhancing growth and biomass accumulation, SA+chitosan showed relatively better performance in improving plant water status and membrane stability, while SA+ascorbic acid contributed more to maintaining photosynthetic performance under drought stress. These results suggest that SA-based treatments may trigger different but potentially complementary physiological responses; however, the study does not provide direct mechanistic evidence to clearly separate pathway-specific effects among the different combinations. Therefore, these mechanistic explanations should be considered tentative and based mainly on observed physiological patterns rather than confirmed causal pathways. Overall, these findings are based on a single ornamental species studied under greenhouse pot conditions; therefore, any extension of the results to other crops or field environments should be made cautiously. Further studies under field conditions and in multiple species, along with more detailed physiological and molecular analyses, are needed to confirm the consistency of these responses under diverse environmental conditions. This is particularly important to address environmental variability, such as soil moisture fluctuations, that cannot be fully replicated under pot conditions.

## Data Availability

All data generated or analyzed during this study are included in this article.
